# Influence of Dentin Priming with Tannin-Rich Plant Extracts on the Longevity of Bonded Composite Restorations

**DOI:** 10.1155/2021/1614643

**Published:** 2021-06-15

**Authors:** Mackeler Ramos Polassi, Thales de Sá Oliveira, Ana Calheiros de Carvalho, Lívia Soman de Medeiros Medeiros, Thiago André Moura Veiga, Carlos Frederico de Oliveira Graeff, Alejandra Hortencia Miranda González, Maria Cristina Marcucci, Simone dos Santos Grecco, Paulo Henrique Perlatti D'Alpino

**Affiliations:** ^1^Universidade Anhanguera de São Paulo, São Paulo, SP, Brazil; ^2^Department of Chemistry, Universidade Federal de São Paulo, Diadema, SP, Brazil; ^3^São Paulo State University (UNESP), School of Sciences, POSMAT—Post-Graduate Program in Materials Science and Technology, Bauru, SP, Brazil; ^4^Triplet Biotechnology Solutions, São Paulo, SP, Brazil; ^5^Science and Technology Institute, Universidade Estadual Paulista—UNESP, São José dos Campos, SP, Brazil

## Abstract

**Objective:**

This *in vitro* study evaluated the influence of bioactive plant extracts as dentin biomodifying agents to improve the longevity of bonded restorations. For that, plant extracts were applied to the dentin surface prior to the adhesive system.

**Materials and Methods:**

Bovine incisors were ground flat to obtain 2 mm thick slices in which conical preparations were made (*N* = 10). Tannin-containing plant extracts were applied to dentin before the application of the restorative system, as follows: control group (untreated, CTL), chlorhexidine 0.12% (CHX), mastruz (*Dysphania ambrosioides,* MTZ), cat's claw (*Uncaria tomentosa,* CTC), guarana (*Paullinia cupana*, GUA), galla chinensis (*Rhus chinensis*, GCH), and tannic acid (extracted from *Acacia decurrens*, TNA). The push-out bond strength test was conducted (0.5 mm/min). Dentin biomodification was assessed by the modulus of elasticity and mass change in bovine tooth sections (0.5 × 1.7 × 7.0 mm). The dentin staining after extract treatments of dentin slices was compared. The dentin surface wettability was also evaluated by means of the contact angles of the adhesive system with the dentin surface and compared with the untreated control group. Data were subjected to ANOVA/Tukey's test (*α* = 0.05).

**Results:**

The bond strength of the restoratives to dentin was not significantly improved by the plant extracts, irrespective of the evaluation time (*p* > 0.05). Except for TNA, the elastic modulus of demineralized dentin significantly reduced after treatment with the plant extracts (*p* < 0.05). The dentin staining correlated with the tannin content of the extracts. The contact angle was significantly reduced when treated with CTC, GCH, and TNA.

**Conclusions:**

The tannin-containing extracts had a questionable effect on the longevity of bonded restorations. The dentin modulus was negatively affected by the extract treatments. Although some of the extracts changed the contact angle, which seems to improve the adhesive monomer permeation, the tannin-rich plant extract application prior to adhesive application was proven to be clinically unfeasible due to dentin staining.

## 1. Introduction

At the tooth-restoration interface occurs an interaction of polymers, collagen, noncollagenous protein, and minerals with the dentin, forming a hybrid layer [1]. The main goal in the bonding of adhesive restorations to dentin is to obtain complete infiltration of the resin monomers into the previously demineralized dentin surface to avoid the degradation of this interfacial area [2]. In this manner, the extent to which the adhesive resin envelops collagen fibrils helps improve the durability of bonding to dentin [3]. Exposed unprotected collagen fibrils after the bonding procedure is regarded to be the main problem that impacts the restoration longevity due to the deterioration that occurs at the hybrid layer [4]. As a consequence, there is a decrease in the bond strength of resin-based materials to dentin, a decrease in the sealing of the restoration margins, and ultimately restoration loss [5].

Different approaches have been proposed to enhance and reinforce the dentin by superficially altering its biochemistry and biomechanical properties [6]. Among these purposes, dentin biomodification is claimed to be a biomimetic method mediated by bioactive agents, which interact with various extracellular components of the dentin matrix, ultimately improving the biomechanics and stability of the dentin collagen matrix [6]. In the process of dentin biomodification, bioactive chemical mediators are regarded to improve the strength of collagen fibrils by forming cross-links [6]. The collagen cross-linking favors the increase of the fibrillar resistance to enzymatic degradation [7] and also provides improved tensile properties [8]. In this manner, synthetic and natural compounds have been proposed to induce collagen cross-linking [9]. Cross-linking is also regarded to maintain the stability of dentin collagen to enzymatic degradation [4]. Inhibitors of matrix metalloproteinases (MMPs) and cysteine cathepsins, which are intensively activated in mildly acidic environments, have been also applied as dentin primers [10, 11]. Tannins, which are polyphenols found in plants, are regarded to inhibit proteases and to present an affinity to dentin collagen, acting as a cross-linking inductor, preventing dentin matrix loss [6, 12].

The present *in vitro* study evaluated the effects of tannin-rich plant extracts on the mechanical properties and degradation of demineralized dentin within a clinically relevant application time. In addition, the push-out bond strength of bonded restorations to dentin was also evaluated after dentin biomodification. The results after 6 months of water biodegradation were compared with those obtained immediately (after 24 h). Additionally, the contact angle measurement of extract-treated dentin formed after adhesive application was analyzed. Extracts from mastruz (*Dysphania ambrosioides*), cat's claw (*Uncaria tomentosa*), guarana (*Paullinia cupana*), galla chinensis (*Rhus chinensis* galls), and tannic acid (obtained from *Acacia decurrens*) were used as dentin biomodifiers. The results were compared with a control, untreated group (negative control) and two positive controls: chlorhexidine 0.12% and tannic acid. The research hypotheses tested were (i) the bond strength of restorations filled with an adhesive restorative system will be negatively influenced by the previous application of plant extracts, irrespective of the evaluation time; (ii) the plant extracts would provide an increase in the elastic modulus of demineralized dentin; and (iii) the plant extracts will significantly affect the contact angle formed between the adhesive system and the dentin surface.

## 2. Materials and Methods

### 2.1. Preparation of Herbal Extracts

The different vegetable drugs (0.750 g) were subjected to extraction at 70°C for 30 min in 150 mL of distillated water (MTZ, CTC, and GUA). The decoction product was completed with distilled water up to 250 mL. Afterward, about 80 mL of the solution was taken and filtered through filter paper, discarding the first 50 mL. The filtrate obtained was called stock solution. The GCH solution was prepared by dissolving the commercially obtained extract in distilled water at a concentration of 4,000 ppm. TNA solution was also prepared in a concentration of 1% (w/v). The experimental groups of the present study are described in Table 1.

### 2.2. Determination of Tannin Levels

To determine the tannin levels, a calibration curve regarding tannic acid was constructed. For that, an aqueous solution of tannic acid, 0.1 mg/mL, was prepared. Then, aliquots (0.5, 1.0, 1.5, 2.0, 2.5, and 3.0 mL) were transferred to 100 mL volumetric balloons. The Folin–Ciocalteu phenol reagent (5 mL) was then added to 10 mL of the aqueous solution of sodium carbonate, and the volume was completed with distilled water. After 30 min (with standard temperature and lighting), each sample was read in the spectrophotometer at 760 nm. After the dilutions, the final concentrations were 0.5, 1.0, 1.5, 2.0, 2.5, and 3.0 *μ*g/mL. The absorbance readings obtained at different concentrations of tannic acid were used to plot the calibration curve. Distilled water was used as a blank solution.

To determine the tannin levels in the plant extracts, the total phenol was initially quantified. For that, 1 mL of the extract was transferred to a 100 mL volumetric balloon containing 50 mL of distilled water. In the same way as employed for the construction of the curve, add 5 mL of the Folin–Ciocalteu phenol reagent, then add 10 mL of the aqueous solution of sodium carbonate, and complete the volume with distilled water. After 30 min, the extracts were read in the spectrophotometer at 760 nm. The residual phenol quantification was also evaluated. Casein (1.0 g) was transferred to a 50 mL Erlenmeyer flask, adding 6 mL of the extract and 12 mL of distilled water. After 3 h under agitation at room temperature, the extracts were filtered and read. For the total phenol quantification, this protocol was repeated. To estimate the tannin levels, the difference between the total phenol level and the noncomplex residual phenol level was calculated [13].

### 2.3. UPLC/DAD-ESI/HRMS/MS Analytical Conditions

Samples (10 *μ*L) from the different extracts were subjected to UPLC/DAD-ESI/HRMS/MS analysis. Thus, a Shimadzu chromatographic system, fitted with the Kinetex 2.6 m C_18_ column and kept in the oven at 55°C, was used. The analytical conditions were set as an exploratory gradient, starting with a low concentration of strong solvent, 15% (acetonitrile HPLC grade, with 1% of formic acid), up to 95% in 12 minutes. The concentration remained constant for 4 minutes, returned to the initial conditions after 1 min, and kept constant for 4 min. The flow was set as 0.4 mL·min^−1^ and remained constant. The UPLC effluent was electrospray ionized and analyzed in positive and negative modes on the high-resolution mass spectrum, fitted with a QToF mass analyzer (Bruker Daltonics, Billerica, MA, USA). The drying gas temperature was defined as 200°C at a flow of 9 L·min^−1^, 2 bars for nebulizer pressure, and 4500 capillary voltage (kV). To characterize the compounds, chromatographic band fragments with a mass charge ratio (*m/z*) between 50 and 1200 da were selected. Sodium formate was used as a calibrator. Data were treated using DataAnalysis 4.4 software (Bruker), and extracted ion chromatograms (EIC) were generated using Target Analysis 1.3 software and an *in-house* Excel list of target candidates, with the compound names and molecular formulas, according to the chemical composition literature for each plant species and/or genus [14–19].

### 2.4. Compressive Bond Strength Test

Seventy bovine incisors were selected, cleaned, and stored in a 0.5% chloramine T solution at 4°C. The specimens were ground flat to obtain 2 mm thick slices as previously described [20]. The specimens were randomly distributed into 6 groups (*n* = 10) according to the dentin biomodification treatment: (1) control (CTL, distilled water), (2) chlorhexidine 0.12% (CHX), (3) mastruz (MTZ), (4) cat's claw (CTC), (5) guarana (GUA), (6), galla chinensis (GCH), and (7) tannic acid (TNA).

Conical preparations were performed in the specimens with margins in dentin (top diameter 4.0 mm, bottom diameter 3.0 mm, 2.0 mm thick). The dentin slices were acid-etched for 15 s with 35% phosphoric acid (Scotchbond Etchant; 3M ESPE, St. Paul, MN, USA) and rinsed for 20 s. The plant extracts were applied for 60 s using a microbrush. The excess solution was dried with absorbent paper. Then, the adhesive system (lot #N808310, Adper Single Bond 2; 3M ESPE, St. Paul, MN, USA) was applied and cured for 10 s. The specimens were positioned over a polyester strip. The composite (lot #18177008007, Filtek Z350XT; 3M ESPE, St. Paul, MN, USA) was inserted into the preparations. The preparations were filled with the composite and then covered with a Mylar strip and then compressed with a glass slide. The composite was then photoactivated for 20 s (1200 mW/cm^2^, Bluephase; Ivoclar Vivadent, Schaan, Liechtenstein). The specimens were then stored at 37°C in physiological saline solution for 24 h. Restorations were finished and polished using abrasive discs (Sof-Lex; 3M ESPE, St. Paul, MN, USA).

The compressive push-out bond strength of bonded restorations was evaluated according to the following factors: (1) dentin pretreatment at two levels: control, untreated dentin, and dentin biomodification with plant extracts and (2) storage time at two levels: immediate (24 h) and after 6 months. Seven experimental groups were categorized (*n* = 10). The push-out bond strength test was conducted as previously described [20], at a crosshead speed of 0.5 mm/min. The compressive force of the probe was applied at the bottom surface of the restoration. The means were expressed in MPa [20]. After the test, a fractographic analysis was also performed at 5× magnification using a dissecting microscope (Stereozoom; Bausch & Lomb, Rochester, NY, USA) according to a previously described classification [20].

### 2.5. Modulus of Elasticity of Demineralized Dentin after Dentin Biomodification

Specimens were obtained as previously described [21, 22]. Briefly, sections of bovine incisor teeth were obtained (0.5 × 1.7 × 7.0 mm) using a water-cooled rotating diamond wheel (IsoMet; Buehler Ltd., Evanston, IL, USA). The specimens were then immersed in 10% phosphoric acid solution (LabChem, Pittsburgh, PA, USA) for 5 h and then thoroughly rinsed with distilled water for 10 min. The specimens were then immersed in distilled water for baseline measurements. Before the treatment, the initial dry weight and initial flexural modulus (three-point bending test) were assessed as previously described [21]. The demineralized specimens (*n* = 15) were treated with plant extracts for 60 s [23]. Then, the specimens were vigorously rinsed with distilled water for 30 s. The flexural modulus was reassessed immediately after immersion, and the treated beams were individually stored in 1 mL of artificial saliva (pH 7.4), containing HEPES 5 *μ*M, CaCl_2_ 2.5 mM, ZnCl_2_ 0.05 mM, and NaCl 120 mM, at 37°C for four weeks to undertake collagen degradation [24].

The specimens were tested on three-point bending setup in a universal testing machine (Instron 4484; Instron Inc., Canton, USA), with a 5 N load cell at 0.5 mm/min crosshead speed. Load-displacement curves were converted to stress-strain curves. The width and thickness of the specimens were measured, and the elastic modulus was calculated at 3% strain as previously described [21]. Data were expressed in MPa, and the percentage variation in elastic modulus was calculated as the ratio of the final value (after treatment with plant extracts) to the initial values (baseline).

### 2.6. Mass Weight Change of Demineralized Dentin after Dentin Biomodification

Demineralized dentin beams were weighted before (*M*_1_) and after (*M*_2_) dentin biomodification with an analytical balance (0.00001 mg precision; Ohaus Analytical Plus, Florham Park, NJ, USA). An additional mass weight assessment was performed after 4 weeks of degradation in artificial saliva (*M*_3_). In each period, the specimens were initially dried in a vacuum desiccator containing silica gel beads for 72 h at room temperature. Mass weight variation (*W*_*mc*_%) was determined as the percentage of gain or loss in mass for each specimen, as previously described [21], based on the following formula for biomodification:

(1) $$\begin{array}{c} W_{mc}\left(\%\right)=\left[\frac{M_{2}\times 100}{M_{1}}\right]-100, \end{array}$$

where *M*_1_ is the demineralized dentin beam mass weight before dentin biomodification and *M*_2_ is the mass weight of the biomodified dentin matrix. Moreover, to assess the biodegradation percentage mass weight variation, the following formula was used:

(2) $$\begin{array}{c} W_{de}\left(\%\right)=\frac{\left[\left(M_{3}-M_{1}\right)\times 100\right]}{M_{1}}, \end{array}$$

where *M*_1_ is the demineralized dentin beam mass weight before dentin biomodification and *M*_3_ is the mass weight of dentin matrix after four weeks of artificial saliva immersion.

### 2.7. Surface Wettability Assessment

Two-millimeter-thick dentin slabs were obtained from bovine incisor teeth by grinding the enamel surface to a 600-grit finish with SiC paper until flat dentin areas were obtained (around 5 mm of width × 6 mm of length). The smear layer was kept intact for the contact angle measurements. The specimens were immersed in distilled water until analysis. As previously described, dentin slices were acid-etched for 15 s with 35% phosphoric acid and water-rinsed for 20 s. The plant extracts were applied for 60 s using a microbrush. The excess solution was dried with absorbent paper. Then, one drop (5 *μ*L) of the adhesive system was deposited using a micropipette (Eppendorf, Hamburg, Germany) onto each dentin slice after dentin biomodification with the plant extracts. Digital images of the specimens were obtained by taking horizontal photographic shots at right angle to the long axis of the dentin with a high-resolution (5.0 megapixel) digital still camera, Nikon 90 (Nikon Inc, Japan), attached to a lens (Nikon Medical-Nikkor, 120 mm f/4IF), positioned 30 mm from the specimen. High-quality images in JPEG (Joint Photographic Experts Group) format at maximum quality and without changes were obtained with an 8× magnification. An angular dimension measuring tool was used to measure the contact angle (*θ*) formed by the contact surface of the dentin specimens and the tangent of the adhesive drop (https://www.ginifab.com/feeds/angle_measurement/). Right and left angle measurements (*n* = 3) were obtained, and then an average was calculated for each specimen. A control, untreated group was also evaluated.

### 2.8. Statistical Analysis

Means and standard deviations of the compressive bond strength test were calculated and statistically analyzed with two-way ANOVA and Tukey's post hoc test, with a preset alpha of 5%. Data of elastic modulus, mass weight variation, and contact angle analysis were statistically analyzed with ANOVA/Tukey's test (5% of significance).

## 3. Results

In Table 2 are displayed the tannin levels for different studied species, illustrating the individual variations. In general, tannin levels can be ranked as follows: GCH > TNA > GUA > CTC > MTZ (Table 2).

**Figure 1:**
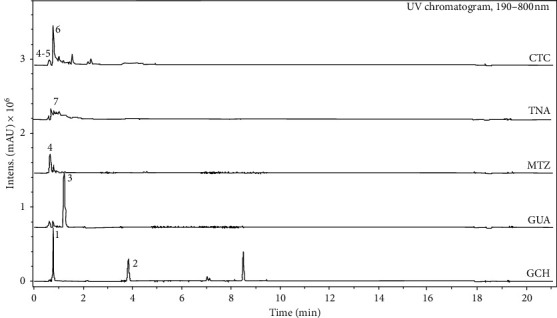
Phytochemical profile (fingerprint) of the plant extracts through hyphenated technique (UPLC/DAD-ESI/HRMS/MS).

Chromatograms of each plant extract are presented in Figure 1, while the compounds identified within the respective retention times (*R*_*t*_), molecular ion masses (*m*/*z*), and relative peak area (RPA) are summarized in Table 3.

Gallic acid (*R*_*t*_ = 0.8 min; *m/z* 169.0139 − [M − H]^−^; RPA = 36.6%) and methoxybenzoic acid derivative (*R*_*t*_ = 3.9 min; *m/z* 151.0570 − [M − H]^−^; RPA = 26.8%) were identified at GCH and caffeine (*R*_*t*_ = 1.3 min; *m/z* 195.0881 − [M + H]^+^; RPA = 84.3%) at GUA. At MTZ were observed ions of organic acids—malic and citric acids (*R*_*t*_ = 0.7 min; *m/z* 133.0145 and 191.0193 − [M − H]^−^; RPA = 77.7%), and those organic acids were also observed at CTC, within quinic acid (*R*_*t*_ = 0.7 min; *m/z* 191.0562 − [M − H]^−^; RPA = 10.5%); however, the major compound identified at CTC was chlorogenic acid (*R*_*t*_ = 0.9 min; *m*/*z* 353.0879 − [M − H]^−^; RPA = 58.0%). Tannic acid presents a molecular weight higher than the upper limit of MS detector specification (*m/z* > 1200). Although it was not possible to observe the molecular ion, some characteristic fragmentation ions were observed for TNA at negative mode—*m/z* 169.0147, 369.0290, and 673.0898 [25].

**Figure 2:**
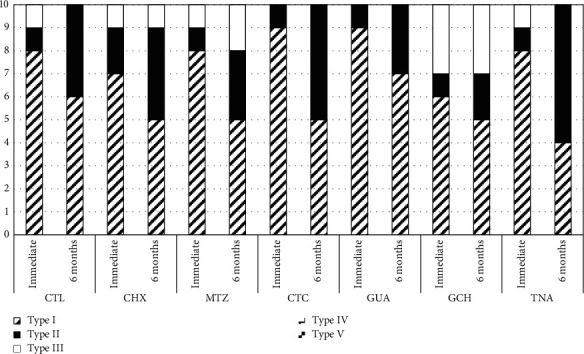
Incidence of failure modes (%) according to the experimental groups.

The results of the push-out bond strength test are described in Table 4. The highest push-out bond strength mean was observed for CTC after 6 months (42.8 MPa), and the lowest when treated with CHX was evaluated after 24 h (34.2 MPa). No significant differences were observed among the experimental groups, regardless of the dentin treatment and the evaluation time (*p* > 0.05). Type 1 failure mode (adhesive mode) was the most frequent, irrespective of the factors evaluated (Figure 2).

**Figure 3:**
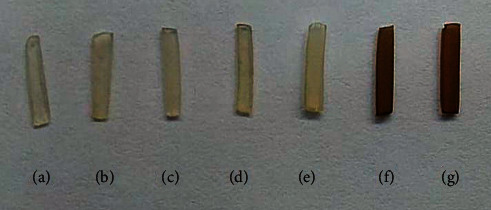
Color changes in demineralized dentin specimens after biodegradation in artificial saliva for four weeks. (a) CTL; (b) CHX; (c) MTZ; (d) CTC; (e) GUA; (f) GCH; and (g) TNA.

Table 5 shows the mean dentin elastic modulus and the standard deviation. The statistical analysis demonstrated significant differences between the TNA-treated group and the experimental groups (*p* < 0.05). TNA induced an increase of 70.8% of the dentin elastic modulus. Except for dentin specimens treated with TNA, the modulus significantly decreased after dentin treatment with the extract plants (from −2.2% for CTC to −49.4% for CHX). After biodegradation in artificial saliva, a significant increase in the elastic modulus was observed for the groups treated with GCH and TNA compared with the control groups (CTL and CHX) and to the other dentin treatments (*p* < 0.05). There was a significant increase of the mass for the experimental groups treated with CHX, MTZ, GUA, and GCH (Table 6). After biodegradation in artificial saliva, all experimental groups exhibited dentine mass reduction, which was significantly higher for the CTL group and for the groups treated with MTZ, CTC, and TNA (Table 6). Representative pictures of the specimens after biodegradation are presented in Figure 3. Darker color was observed in specimens treated with GCH and TNA, whereas those treated with the other extracts demonstrated reduced, but with clear signs of pigmentation.

Contact angles of the adhesive with the extract-treated dentin are demonstrated in Table 7. The highest contact angle was observed in the CTL group (38.0 degrees), and the lowest (20.7 degrees) was observed when TNA was applied previously to the adhesive system. The application of CTC, GCH, and TNA significantly decreased the contact angle compared with the control group (*p* < 0.05).

## 4. Discussion

The use of biomodifying agents was proposed to provide stabilization and strengthening of the collagen matrix by improving the mechanical properties of the dentin [6]. In this manner, these agents are regarded to increase the bond strength of bonded restorations to dentin, to reduce the biodegradation rate of demineralized collagen, and consequently, to increase the longevity of adhesive restorations [4, 26]. Cross-linking agents are claimed to react in different ways with collagen, creating different kinds and extents of cross-links, mainly via bonding with hydrogen ions (with natural agents) and covalent bonds (with synthetic agents) [6, 27]. Plant extracts have also been used to induce dentin biomodification mainly due to the presence of tannins, especially proanthocyanidins [28]. Tannins are polyphenolic compounds that are associated with numerous secondary activities in plants [29]. Tannins can be used as a potential cross-linker for the development of biomaterials for clinical applications [30]. Among several actions, tannins were found to have an affinity for type I collagen [8], the most abundantly found in dentin, which is exposed after acid conditioning. Tannins are regarded to bind to collagen, inducing significant changes in the conformation of the triple helix of type I collagen, thus increasing its structural, thermal, and enzymatic stability [8].

The plant drugs selected in the present study presented complex mixtures of compounds (Figure 1). Phenols and polyphenols such as gallic acid, methoxybenzoic derivative, and chlorogenic acid, found in plant materials, were detected in the extracts of GCH and CTC. While caffeine was identified in GUA extract, other organic acids such as malic, citric, and quinic acids were identified in MTZ and CTC. TNA, a commercial form of condensed tannin, is a naturally occurring collagen cross-linking agent consisting of a complex mixture of polygalloylglucose esters with weak acidity [31, 32]. Although most studies in which the biomodifying action of dentin with plant drugs was evaluated focused on the presence of tannins, other bioactive agents with lower molecular weight can also act as biomodifiers, inducing collagen cross-linking [26].

The dentin biomodification effect of bioactive agents found in the plant extracts had no influence on the bond strength of bonded restorations to dentin, regardless of the evaluation period (*p* > 0.05). In this manner, the first hypothesis, which anticipated that the bond strength to dentin would be negatively influenced by the previous application of extract plants, was not accepted. Conversely, except when treated with TNA, the mechanical properties of exposed dentinal collagen were negatively influenced by all dentin treatments with the plant extracts (*p* > 0.05). Thus, the second hypothesis in which it was speculated that the extract plants would provide an increase in the elastic modulus of demineralized dentin was not accepted.

Tannin-rich extracts as cross-linkers prior to the application of adhesive restorative materials demonstrated superior results when compared with chlorhexidine [33–36]. Conversely, concerns have been expressed over their actual benefits, especially when cross-linkers are applied in clinically relevant application times [27]. It has been questioned that the ability of a cross-linking agent to react with the dentin matrix to avoid the degradation of collagen at the adhesive interfacial area depends on its chemical and structural characteristics [27]. In the present study, only TNA proved to be equally effective in chemically modifying collagen, which explains the reason to use it as a positive control. A previous study demonstrated that TNA is able to reduce the enzymatic degradation to improve the mechanical properties of dentin, thus increasing the bond strength of resin-based restorations to dentin [32].

The fact that the plant extracts reduced the modulus of elasticity after treatment, in a certain way, demonstrated that these extracts were not able to induce collagen cross-linking. Although the compounds found in tannin-rich plant extracts seem to present multiple phenolic functionalities, oligomeric structures that have high biomodifying potential may present higher molecular weight [37]. This helps explain their slower ability to diffuse in the collagen matrix, explaining the lesser influence on the elastic modulus and resistance to degradation when applied for 1 minute [23]. It has been also claimed that changes in the elastic modulus induced by the plant extracts should be associated with its ability to nucleate calcium and phosphate to form amorphous calcium phosphate crystals to remineralize the exposed uninfiltrated collagen [3]. Thus, it can be speculated that the true biomodifying action of the extracts after treatment of the dentin fragments should be evaluated after immersion in a saturated solution of calcium and phosphate (Ca/P ratio = 1.67) [3]. This fact can be corroborated by the “late” biomodifying action of galla chinensis, which induced an increased elastic modulus after immersion in calcium-containing artificial saliva.

The interactions and complexation of the compounds of the extracts with dentin collagen also seem to explain the variations in mass (gain or loss) observed in the present study (Table 4). In addition, the resistance to biodegradation is related not only to the increase in the modulus of elasticity induced by the formation of collagen cross-linking but also to the hydrophilicity of the biomodifying agents [38]. This also explains the stability of the bond strength in the experimental groups after 6 months. In a previous study, it was highlighted that the ability of the plant extracts to dehydrate collagen fibrils by means of the cross-linking effect would render a more promising collagen substrate for hybridization and eventually a more stable hybrid layer [39]. On the other hand, it has been pointed out that hydrophobic bioactive agents can also induce less degradation of the collagen matrix when compared with hydrophilic agents [23]. Biomodifying agents were also regarded to induce the formation of cross-links in the dentin collagen during the formation of chemical bonds between the cross-linking agents and the collagen. It has been advocated that these agents would remove proteoglycans from the dentin matrix, replace the water bound to the collagen fibrils, and reduce its hydrophilicity [38]. In this manner, these agents would favor a greater diffusion of monomers of the adhesive system through the collagen network, allowing it fully expand [40], reducing the rates of biodegradation [4, 6].

In a certain way, the dentin wettability after treatment with the plant extracts, which would improve the permeation of the adhesive system and the longevity of the adhesive interfaces, needs to be further confirmed to certify the action of these biomodifying agents. At a molecular level, the intermolecular spaces between the collagen fibrils are preserved by the water surrounding the triple helices of the collagen molecules [3]. Consequently, hydrogen bridges are formed preventing direct contact between chains, thus avoiding collagen collapse [41]. For the adhesive to have an effective bond to the dentin, it is necessary to have a network structure of noncollapsed collagen fibrils, with interfibrillar spaces filled with water, allowing the proper infiltration of resin monomers in the demineralized intertubular dentin, to then form the hybrid layer [42]. The fact that the plant drugs of the present study are water-soluble seems to have favored the dentin wettability prior to the adhesive system. The contact angle of the adhesive with dentin was significantly reduced when treated with the plant extracts CTC, GCH, and TNA in comparison with the control groups (CTL and CHX). In this manner, the third hypothesis, which speculated that the extract plants would significantly affect the contact angle formed between the adhesive system and the dentin surface, was partially accepted.

In addition, considering the clinical application of the plant extracts in dentin, it was observed that the greatest changes in the color of the dentin fragments correlated with the highest concentration of tannins in them. This in a way makes its clinical use unfeasible. In spite of the favorable results of GCH in remineralizing dental tissues in several studies [43, 44], dental products with this appeal are certainly not available so far because of the dentin color change. It has been widely known that green tea, a popular drink consumed daily by millions of people worldwide, is known to cause blackening of the teeth because it is rich in epigallocatechin gallate (65% of its composition) [45]. This was also observed in another previous study in which a tannin-rich bioactive agent stained the dentin specimens intensely in the dark brown shade [23]. The present study demonstrated that the plant drug extracts tested had different effects on dentin and more specifically on dentin collagen. Different from that of other studies, a questionable effect of the plant extracts with variable concentration of tannins as dentin biomodifiers was demonstrated. Although the extracts of plant drugs had no impact on the bond strength, the dentin modulus was significantly reduced after the dentin treatment with the extract plants. In addition, the use of tannin-rich extracts prior to adhesive application was proven to be clinically unfeasible due to dentin staining. The highest tanning content in the extracts induced darker staining, which is favored by the tubular structure of the dentin, diffused by capillarity through other regions of the dentinal structure.

Considering its limitations, the present study was conducted *in vitro* with the extracts. In addition, it was not clear how long is the duration of the contact of the tannins and other compounds of plant extracts with the dentin collagen fibrils. This could influence the binding mechanism of tannins to collagen fibrils and their potential stabilization mechanism [3]. In spite of these limitations, these results provided important new information regarding the application of these plant extracts previously to the adhesive bonding. Furthermore, the results of a previous study corroborate with our results, which proved that tannic acid (TNA) affects the mechanical properties of demineralized dentin by increasing its stiffness and provides collagen stabilization [32]. Future studies are necessary to provide more information, including the evaluation of the effect of these tannin-rich plant extracts on the nanostructure of collagen fibrils and *in vivo* studies. In spite of the clinically negative impact of dentin staining, some of the plant extracts were able to change the dentin wettability, as demonstrated by the reduction in contact angle. This seems to favor a greater diffusion and permeation of the resinous monomers of the adhesive throughout the collagen fibrils.

## 5. Conclusions

Within the limitations of this study, it can be concluded that although the bioactive plant extracts had no impact on the bond strength to dentin regardless of the evaluation time, it was observed a negative influence on the apparent modulus of elasticity of the demineralized dentin. Some of the extracts changed the contact angle of the adhesive with the dentin, possibly improving the adhesive permeation throughout the demineralized dentin. Conversely, the higher the tannin content, the more the extracts stained the dentin, which is impeditive for its clinical application. Further studies are needed to understand the mechanisms involved in the intimacy of the tooth-restoration interface when using the evaluated plant drug extract.

## Figures and Tables

**Table 1:** Vegetable drugs selected for the present study.

Popular name	Scientific name	Source	Lot#
Mastruz (MTZ)	*Dysphania ambrosioides*	Leaves, bark, and stems	1737826333-8 Panizza Laboratório São Paulo, Brazil
Cat's claw (CTC)	*Uncaria tomentosa*	Leaves, bark, and stems	9816253436-3 Panizza Laboratório São Paulo, Brazil
Guarana (GUA)	*Paullinia cupana*	Dry seeds	1589538295-5 Panizza Laboratório São Paulo, Brazil
Galla chinensis (GCH)	*Rhus chinensis*	Dried extract	GCE99%-20180628 Changsha Vigorous-tech Co. Ltd. Hunan, China
Tannic acid (TNA)	Obtained from *Acacia decurrens*	Purified fraction	34783 Dinâmica laboratório São Paulo, Brazil

**Table 2:** Extracts analyzed with respect to tannin level (% w/w).

Extract	Tannin (%)
MTZ	**0.0** (0.0)
CTC	**9.5** (0.9)
GUA	**10.8** (0.5)
GCH	**33.7** (0.6)
TNA	**26.2** (2.6)

Values within the parenthesis are standard deviation.

**Table 3:** Major compounds identified in plant extracts with UPLC/DAD-ESI/HRMS/MS.

#^*∗*^	Compound name	*R*_*t*_ (min)	*m/z* (Da)	RPA (%)	Extract
1	Gallic acid	0.8	169.0139	36.6	GCH
2	Methoxybenzoic acid derivative	3.9	151.0570	26.8	GCH
3	Caffeine	1.3	195.0881	84.3	GUA
4	Malic acid	0.7	133.0145	77.7	MTZ; CTC
	Citric acid	0.7	191.0193		
5	Quinic acid	0.7	191.0562	10.5	CTC
6	Chlorogenic acid	0.9	353.0879	58.0	CTC
7	Tannic acid	—	>1200	—	TNA

^*∗*^Number of compounds identified in Figure 1. Rt: retention time; RPA: relative peak area.

**Table 4:** Push-out strength means as a function of the evaluation time.

	CTL	CHX	MTZ	CTC	GUA	GCH	TNA
24 hours	39.1^a.A^ (15.7)	34.2^a.A^ (11.8)	41.1^a.A^ (20.8)	42.0^a.A^ (18.5)	35.3^a.A^ (15.3)	39.9^a.A^ (13.7)	42.6^a.A^ (9.4)
6 months	36.2^a.A^ (14.0)	34.8^a.A^ (18.1)	41.7^a.A^ (12.5)	42.8^a.A^ (10.5)	39.4^a.A^ (10.9)	40.2^a.A^ (6.3)	40.0^a.A^ (8.2)

Mean values followed by different capital letters in row and small letters in column indicate significant differences (*p* < 0.05). Values within the parenthesis are standard deviation.

**Table 5:** Means (standard deviation) of modulus of elasticity (in MPa) of demineralized dentin as a function of the treatment.

Experimental groups	Initial modulus (MPa)	Elastic modulus variation after dentin biomodification (%)	Elastic modulus variation after biodegradation in artificial saliva (%)
CTL	2.04^A^ (0.95)	−27.5^B^ (32.2)	−40.7^B^ (25.5)
CHX	2.81^A^ (1.12)	−49.4^B^ (29.4)	−37.1^B^ (47.5)
MTZ	3.15^A^ (2.22)	−34.7^B^ (47.8)	−50.0^B^ (44.3)
CTC	2.14^A^ (0.96)	−2.2^B^ (51.0)	−28.1^B^ (31.3)
GUA	2.39^A^ (1.52)	−21.4^B^ (29.7)	−28.7^B^ (32.3)
GCH	2.45^A^ (1.36)	−3.9^B^ (41.1)	6.8^A^ (32.3)
TNA	2.41^A^ (1.38)	70.8^A^ (87.9)	27.6^A^ (72.1)

Different capital letters (A and B) for comparisons between lines indicate significant difference (*p* < 0.05). *N* = 15.

**Table 6:** Mean (SD) of mass variation (%) of dentin specimens as a function of the treatment.

Experimental groups	Mass variation (%) after dentin biomodification	Mass variation (%) 4 weeks of degradation
CTL	−4.8 (6.3)^B^	−16.8 (6.9)^B^
CHX	+7.2 (3.6)^A^	−7.0 (6.0)^A^
MTZ	+0.7 (13.4)^B^	−10.0 (14.8)^B^
CTC	−0.6 (3.9)^B^	−12.2 (4.9)^B^
GUA	+3.3 (3.9)^A^	−7.8 (5.2)^A^
GCH	+1.0 (4.6)^B^	−4.9 (6.1)^A^
TNA	−2.5 (2.8)^B^	−8.5 (13.6)^B^

Different letters within each column indicate statistically significant difference (*p* < 0.05). *N* = 15.

**Table 7:** Contact angle formed by the adhesive system on dentin treated with different plant drug extracts.

Experimental groups	Contact angle^*∗*^
CTL	38.0 (2.0)^A^
CHX	31.3 (0.3)^A^
MTZ	36.3 (0.3)^A^
CTC	25.8 (0.2)^B^
GUA	29.2 (1.2)^A^
GCH	24.7 (0.7)^B^
TNA	20.7 (0.3)^B^

Different letters (A and B) within the columns indicate significant difference (*p* < 0.05). ^*∗*^In degrees.

## Data Availability

The data used to support the findings of this study are available from the corresponding author upon request.
